# Cortisol Awakening Response and Walking Speed in Older People

**DOI:** 10.1371/journal.pone.0152071

**Published:** 2016-05-18

**Authors:** Matias M. Pulopulos, Sara Puig-Perez, Vanesa Hidalgo, Carolina Villada, Alicia Salvador

**Affiliations:** Laboratory of Social Cognitive Neuroscience, Department of Psychobiology and IDOCAL, Universitat de València, Valencia, Spain; University of Marburg, GERMANY

## Abstract

In older people, less diurnal variability in cortisol levels has been consistently related to worse physical performance, especially to slower walking speed (WS). The cortisol awakening response (CAR) is a discrete component of the hypothalamic-pituitary-adrenal axis that has been related to several health problems, such as cardiovascular disease and/or worse performance on executive function and memory. The relationship between the CAR and physical performance in older people is poorly understood. In this study, in 86 older people (mean age = 64.42, SD = 3.93), we investigated the relationship between the CAR and WS, a commonly used measure of physical performance in the older population that has also been related to health problems, such as cardiovascular disease and executive function performance in older people. Additionally, we studied whether the relationship between the CAR and WS was independent from cortisol levels on awakening and several possible confounders. Results showed that a CAR of reduced magnitude (measured with 3 samples each day, for two consecutive days, and calculated as the area under the curve with respect to the increase), but not cortisol levels on awakening, was related to slower WS. In addition, this relationship was independent from cortisol levels on awakening. It is possible that a CAR of reduced magnitude would contribute to less diurnal cortisol variability, affecting physical performance. Additionally, it is possible that a CAR of reduced magnitude affects WS through a possible negative effect on executive function, or that the association between the CAR and WS is due to the fact that both are related to similar health problems and to changes in cognitive performance in older people.

## Introduction

The activity of the hypothalamic-pituitary-adrenal axis (HPA-axis) and the change in cortisol levels follow a circadian rhythm. This circadian rhythm involves three discrete components: (i) the cortisol awakening response (CAR), (ii) a decline in cortisol levels during the rest of the day (showing a sharp decline in the morning and a gradual decline in the afternoon and early evening); and (iii) an increase in cortisol levels from the second half of the night until waking [1]. The CAR consists of a rapid 50 to 160% increase in cortisol concentration after awakening that typically peaks between 30 and 45min later [2]. A dysregulation of the CAR, independently from the rest of the diurnal HPA-axis activity, has been related to several health problems. Thus, a CAR of reduced magnitude (i.e., a reduced cortisol increase post-awakening) has been related to chronic stress, cardiovascular disease, sleep disorders (see [1]), and worse performance on frontal cortex-related cognitive tasks in older people [3, 4]. Additionally, a CAR of increased magnitude has been observed in men with visceral obesity, patients with upper respiratory illnesses, and women with borderline personality disorder (see [1]), and it has been related to worse performance on hippocampus-related memory tasks in older people [3].

Walking speed (WS) is an objective and commonly used measure of physical performance in older people [5] that has also been related to worse cognitive performance (especially executive function) and other health problems (e.g., cardiovascular disease) in older people [6]. Importantly, recent studies have shown that individuals who walk slower show less variability in diurnal HPA-axis activity (i.e., lower cortisol levels in the morning and higher cortisol levels in the evening) (e.g., [7, 8, 9, 10]), and that an HPA-axis dysregulation might affect physical performance, including WS [8]. A possible explanation for the relationship between the HPA-axis and WS is that a dysregulation of the HPA-axis might be a potential mechanism for sarcopenia (i.e., loss of muscle mass and strength), affecting physical performance and WS [8]. However, in contrast to the well-established relationship between WS and diurnal HPA-axis activity, the specific relationship between the CAR and WS is not fully understood. In this regard, a CAR of reduced magnitude has shown a weak association with slower WS [8], faster WS [7], or even no association with WS [10]. Importantly, Gardner et al. [9] performed an individual-participant meta-analysis that included data from six middle-aged and older adult cohorts from population-based studies to investigate the relationship between HPA-axis activity and WS [9]. The relationship between CAR and WS was explored in three out of the six cohorts, containing participants included in [7] and [8], but not in [10]. The authors concluded that there is an association between a CAR of reduced magnitude and slower WS in older people [9]. Overall, conflicting evidence has been reported, and more research is needed to confirm the relationship between CAR and WS observed in Gardner et al. [9].

Although all these previous studies used large sample sizes and had high statistical power, it is possible that some methodological issues could underlie these mixed results [7–10]. In this regard, these studies used only two salivary samples (awakening and 30min later) to measure the CAR. Given that the CAR peaks between 30min and 45min after awakening, the inclusion of only one saliva sample at 30min may have affected the CAR measurements. Moreover, specifically in Kumari et al [7] and Johar et al., [10], the CAR was measured on only one day, which could also affect the reliability of the CAR measurements [2]. Additionally, none of these studies controlled for the perceived stress of the individuals during the previous months, an issue that has been associated with an increased CAR [11]. Finally, it has been shown that low cortisol levels at awakening (an indicator of pre-awakening cortisol secretion) are related to a CAR of increased magnitude, and that it is a measure that should be studied independently from the CAR [2]. Therefore, it is important to investigate whether the possible relationship between the CAR and WS is independent from cortisol levels on awakening.

Therefore, we aimed to investigate whether the CAR (measured with 3 samples each day, for two consecutive days) was related to WS in older people, and whether this relationship was independent from cortisol levels on awakening. Additionally, we controlled for the possible effects of several possible confounders. Based on the results from Gardner et al. [9] (an individual-participant meta-analysis), we expected a CAR of reduced magnitude to be related to slower WS.

## Method

### Participants

Eighty-eight participants who could ambulate household distances independently were recruited for this study. After the exclusion of two participants who were outliers for cortisol data, the final sample was composed of 86 participants from 56 to 72 years old (men = 41; women = 45) (see Table 1 for sample characteristics). The exclusion criteria were: alcohol or other drug abuse, endocrine diseases that affect HPA-axis activity (e.g., Cushing’s syndrome), psychiatric illness, using medication that was able to influence hormonal levels (e.g., glucocorticoids or antidepressants), and having been under general anesthesia once or more in the past year. All the female participants were postmenopausal, had had their last menstrual period more than 2 years before the testing time, and were not receiving treatment for hormone replacement. Results of the Spanish version of the Mini-Mental Status Examination [12] indicated the absence of cognitive impairment. The Ethics Committee of the University of Valencia approved the protocol, and all the participants provided written informed consent. This study was conducted according to the principles expressed in the Declaration of Helsinki.

**Table 1:** Characteristics of the sample.

	Mean±SD
Age (years)	64.42±3.93
Educational level	2.71±1.10
SES	5.51±1.27
BMI (kg/m^2^)	27.17±3.41
Physical activity	1.79±0.74
PSS	16.17±6.41
Walking speed test (sec.)	8.00±1.07
Sleep hours (hh:mm)	06:41±00:54
Wake-up time (hh:mm)	07:07±00:51
Stress_DayBefore_	1.99±0.80
Stress_SameDay_	2.17±0.87
Sleep quality	3.49±0.79
CAR	239.34±151.81
Cortisol Awakening (nmol/L)	7.11±2.99
Cortisol 30min (nmol/L)	15.12±6.51
Cortisol 45min (nmol/L)	14.98±6.60

SES: Subjective socioeconomic status; BMI: Body Mass Index; PSS: Perceived Stress Scale; CAR (calculated as the AUCi using cortisol levels on awakening, +30min and +45min): Cortisol awakening response. Educational level (range: 0 = no studies, 1 = primary school, 2 = secondary education, 3 = university and higher education, 4 = postgraduate). Physical activity (range: 0 = none; 1 = low; 2 = moderate; 3 = high). Stress_DayBefore_: stressfulness of the day preceding saliva sample collection (1 = Not at all, to 5 = Extremely). Stress_SameDay_: expected stressfulness of the same day of saliva sample collection (1 = Not at all, to 5 = Extremely). Sleep Quality: sleep quality of the night preceding the saliva sample collection (1 = Quite poor, to 5 = Quite good).

### Measurements

#### Walking Speed

The time needed to walk a short distance was used as a measure of walking speed. Participants were asked to walk 10m on a line at their usual speed, turn around, and walk back as fast as possible without running. The time taken to walk the six central meters in both directions was measured using a stopwatch (higher scores indicate a worse performance). Previous studies have shown the reliability of this task in older populations [5, 13].

#### Cortisol measurements

After the WS test, the participants collected a total of 3 samples each day, for two consecutive weekdays. Saliva samples were collected to measure the CAR (using salivettes Sarstedt, Nümbrecht, Germany) immediately after waking (cortisol awakening) and 30min and 45min post-awakening. Additionally, participants logged the time of each saliva collection, their sleep duration the nights before the CAR measurements, and their awakening time. Participants also had to answer questions about state stress and sleep quality (see section 2.2.3. for a more detailed description). Salivettes were stored in MEMS TrackCap containers (MEMS 6 TrackCap Monitor, Aardex Ltd., Switzerland) to objectively verify whether the second and third salivary samples were performed 30min and 45min after participants performed the first salivary sample (immediately post-awakening). Participants were thoroughly instructed about how to provide the saliva samples. A demonstration was made by the experimenter, and participants were given written instructions. The instructions were as follows: (i) place the tubes and the MEMS TrackCap containers, the written instructions, and the log near your bed so you do not have to stand up to provide the first saliva sample; (ii) to provide the saliva sample take a cotton swab and place it in your mouth for 2 min and move it around while chewing it slightly; (iii) after you have provided the first saliva sample you can stand up and move around; (iv) you cannot drink (except water), eat, brush your teeth, take any medication or do any physical exercise until you have finished the three saliva samples. Additionally, the following information was provided to the participants in order to maximize adherence to the protocol: (i) Participants were informed about the importance of collecting the awakening sample immediately on awakening; (ii) the meaning of “the moment of awakening” was defined and explained to participants; (iii) participants had the mobile phone number of a researcher from the group to ask any questions during the salivary sampling process; (iv) participants were informed that their sampling accuracy would be monitored; (v) salivettes were differentiated with clear labels; and (vi) participants were asked not to start the saliva sampling collection if they thought there had been a delay of more than 5min between the awakening time and the first salivary sample. After providing the saliva samples, participants stored their samples in their home fridge and brought the samples to the university within three days after completion. There was a mean of 12 days (±1.31) between the WS test and the measurement of the cortisol levels at home.

Once in the lab, the samples were centrifuged at 3000 rpm for 5 min, resulting in a clear supernatant of low viscosity that was stored at 80 C until the analyses were performed in the Central Research Unit (Unidad Central de Investigación) of the University of Valencia (Spain). Salivary cortisol was measured in duplicate, and each participant’s samples were analyzed in the same trial. The samples were analyzed by a competitive solid phase radioimmunoassay (tube coated), using the commercial kit Spectria Cortisol RIA from Orion Diagnostica (Espoo, Finland). Assay sensitivity was 0.8 nmol/L, and the within- and inter-assay variation coefficients were all below 8%.

#### Demographics, health-related characteristics, stress measurements, and sleep quality

Participants filled out a questionnaire on demographic and health-related characteristics (e.g., age, smoking status, physical activity), and they completed the Perceived Stress Scale (PSS; [14]) to assess overall perceived stress in the past month. Additionally, each day, after giving the last saliva sample (i.e., 45min post-awakening), the participants answered questions about stress and sleep quality. Using a 5-point Likert scale, they indicated the stressfulness of the day preceding the saliva sample collection (Stress_DayBefore_: 1 = Not at all, to 5 = Extremely), the expected stressfulness for the same day of saliva sample collection (Stress_SameDay_: 1 = Not at all, to 5 = Extremely), and the sleep quality the night before the saliva sample collection (1 = Quite poor, to 5 = Quite good).

#### Statistical analysis and data management

Data were tested for normal distribution and homogeneity of variance using Shapiro–Wilk and Levene’s tests before the statistical procedures were applied. These analyses revealed that cortisol levels were positively skewed. Following Kobayashi and Miyazaki’s [15] recommendations for cortisol levels measured in the early morning, in this study the cortisol data were square root transformed. As an index of the CAR, we calculated the area under the curve with respect to the increase (AUCi) using cortisol levels on awakening, +30min and +45min (see [16] for the formula). Two women were removed from the analyses because they were outliers for cortisol concentration (+3 S.D).

Some authors have indicated that a delay when performing the first salivary sample is an important cofounder that affects CAR measurements and needs to be controlled [2, 17, 18]. Therefore, using self-reported data and MEMS TrackCap, we identified one participant who performed the first salivary sample more than 5min after awakening on one day of the salivary sampling, and the cortisol data from this suspected non-adherent day were excluded from the analyses.

Nonetheless, self-reported adherence cannot be relied on because the post-awakening time is a period characterized by a state of reduced cognitive and motor performance (i.e., sleep inertia) [19] that is likely to increase the difficulty of performing the first salivary sample immediately after awakening [17]. Thus, if only self-reported awakening data is used to exclude suspected non-adherent participants, it is possible that non-adherent CAR days would still be included in the study, and so more control is needed. Along these lines, previous research has proposed that, in most individuals, a negative CAR (i.e., a decrease in cortisol levels after awakening) may be caused by a delay in performing the first saliva sample [18]. To control for this possible effect, we identified those participants who showed a negative CAR on one or both days. Twenty-five participants (men = 16; women = 9) showed a negative CAR on one day (including the participant who self-reported a delay in the first salivary sample). None of the participants showed a negative CAR on both days. When exploring the differences between days in the pattern of cortisol levels for these two subgroups (i.e., participants with positive CARs on 2 days and participants with positive CAR on only 1 day), results showed that participants with positive CAR on 2 days did not differ in the cortisol levels in each sample across days (all *p*>0.204). Instead, participants with a positive CAR on only one day showed higher cortisol levels at awakening on the day with negative CAR than on the day with positive CAR (*p*<0.001). Additionally, this subgroup also showed lower cortisol levels at +30min and 45+min on the day with negative CAR compared to their cortisol levels on the day with positive CAR (all *p*<0.001). Given that participants in this latter subgroup showed higher cortisol levels at awakening on the day with a negative CAR compared to the day with a positive CAR, the negative CAR might be caused by a delay in taking the sample immediately post-awakening. Thus, for participants showing a positive CAR on only one day, only the data for the positive CAR day were used in the analyses. For participants showing a positive CAR on both days, the CAR data for the two days were averaged. Although this approach does not guarantee the inclusion of only adherent CAR days, it maximizes the possibility of excluding non-adherent CAR days in the analyses. Additionally, some clinical and/or other morbidity symptoms have also been related to blunted or negative CAR [20–22]. Therefore, this method allows us to exclude participants with undiagnosed diseases or other unknown reasons associated with a lack of cortisol increase after awakening.

There were no significant differences between participants showing positive CAR on 1 or 2 days in: age, educational level, subjective Socio-Economic Status (SES; measured using the MacArthur Scale of Subjective Social Status [23]), PSS, walking speed, physical activity, sleep hours, and wake-up time (all *p*>0.521), but participants with a positive CAR on only one day showed slightly higher body mass index (BMI) (*p* = 0.055).

Correlation analyses were performed to investigate the unadjusted relationships between cortisol data, WS, demographics, health related characteristics, stress measures, sleep quality, mean sleep time and time of awakening. Regression analyses were performed to investigate whether the CAR and cortisol awakening were related to WS, controlling for several confounders (e.g., [1, 2]). As covariates, in the first step we included age, BMI, SES, smoking status (0 = No; 1 = Yes), PSS, physical activity (0 = None; 1 = Low; 2 = Moderate; 3 = High), Stress_DayBefore_, Stress_SameDay_, sleep quality, and time of awakening and mean sleep time on the days of saliva sampling. Tolerance values did not indicate a multicollinearity problem for the covariates included in the regression analyses (all tolerance values >0.434). In step 2, we added the CAR or cortisol awakening. The power of the study is 0.82 for a small-to-medium effect size (calculated using G*Power 3.1.9.2 [24, 25]).

## Results

### Correlation analyses

Table 2 shows the results of the unadjusted correlation analyses. Among the most important results, slower WS was related to a CAR of reduced magnitude (*r* = -0.223; *p* = 0.039), lower SES (*r* = -0.227; *p* = 0.035), and less physical activity (*r* = -0.365; *p* = 0.001). Slower WS was also related to older age (*r* = 0.279; *p* = 0.009) and higher perceived stress in the past month (*r* = 0.242; *p* = 0.025). Higher cortisol on awakening was related to younger age (*r* = -0.216; *p* = 0.046), a later awakening time (*r* = 0.318; *p* = 0.003), and higher mean sleep time (*r* = 0.249; *p* = 0.021).

**Table 2:** Unadjusted correlation analyses.

	WS	CAR	Cort Awak.	Age	BMI	SES	Physical Act.	Smoking	PSS	Stress _DayBefore_	Stress _SameDay_	Sleep quality	Awak. time
WS	--													
CAR	-0.223*												
Cort Awak.	-0.142	0.187^#^											
Age	0.279*	-0.081	-0.216*										
BMI	0.166	-0.184^#^	0.067	0.190^#^									
SES	-0.227*	-0.120	0.140	-0.166	-0.118								
Physical Act.	-0.365*	0.021	0.088	-0.197^#^	-0.210^#^	0.003							
Smoking	-0.031	-0.073	-0.056	0.012	-0.094	-0.140	0.071						
PSS	0.242*	-0.025	0.064	-0.007	0.267*	-0.113	-0.174	0.024					
Stress_DayBefore_	0.034	-0.119	0.031	-0.219*	-0.264*	-0.026	0.038	0.159	0.148				
Stress_SameDay_	0.041	0.008	-0.045	-0.190^#^	-0.137	-0.111	0.093	-0.020	0.217*	0.646*			
Sleep quality	-0.143	-0.070	-0.076	-0.226*	-0.185^#^	0.162	-0.097	0.035	-0.179^#^	0.255*	0.163		
Awak. time	0.077	-0.011	0.318*	-0.023	0.023	0.066	0.040	0.184^#^	0.143	0.008	-0.063	-0.013	
Mean sleep time	-0.002	0.058	0.249*	0.016	-0.016	0.080	0.039	0.054	-0.028	0.053	-0.076	-0.153	0.677*

WS: Walking speed; CAR (calculated as the AUCi using cortisol levels on awakening, +30min and +45min): Cortisol awakening response; Cort. Awak.: Cortisol awakening; BMI: Body Mass Index; SES: Subjective socioeconomic status; Physical Act.: Physical Activity; PSS: Perceived Stress Scale; Awak. time: Awakening time.

*p<0.05

^#^p<0.10.

### Regression analyses

Regression analyses, (see Table 3) controlling for possible confounders (age, BMI, SES, smoking status, physical activity, PSS, stressfulness of the day preceding saliva sample collection, expected stressfulness of the same day of saliva sample collection, sleep quality of the night preceding the saliva sample collection, time of waking and mean sleep time the day of saliva sample collection), indicated that a CAR of reduced magnitude was related to slower WS (*p* = 0.022). This association was not significant for cortisol awakening (*p* = 0.257). To explore whether this latter relationship was independent from the cortisol levels on awakening, we included cortisol on awakening in step 1 as a covariate. This analysis showed that a CAR of reduced magnitude was still related to slower WS, regardless of the cortisol awakening concentrations (β = -0.224; *p* = 0.039).

**Table 3:** Regression analyses with cortisol awakening and CAR as predictors and walking speed as dependent variable.

Step 1	β			*p*	
Age	0.191			0.081	
BMI	-0.011			0.920	
SES	-0.170			0.111	
Physical activity	-0.325			0.003	
Smoking status	-0.075			0.482	
PSS	0.091			0.435	
Stress_DayBefore_	0.110			0.444	
Stress_SameDay_	0.021			0.877	
Sleep quality	-0.131			0.257	
Awakening time	0.189			0.198	
Mean sleep time	-0.124			0.393	
Step 2	Adj *R*^2^	Δ*R*^2^	*F*(1,76)	β	*p*
CAR	0.214	0.050	5.438	-0.241	0.022
Cortisol awakening	0.170	0.013	1.303	-0.126	0.257

BMI: Body Mass Index; SES: Subjective socioeconomic status; PSS: Perceived Stress Scale; Adj *R*^2^: Adjusted *R*^2^; Δ*R*^2^: Change in *R*^2^; CAR (calculated as the AUCi using cortisol levels on awakening, +30min and +45min): Cortisol awakening response. *Step 1 (covariates)* = In the first step of the regression analyses, we included as covariates: age, BMI, SES, smoking status (0 = No; 1 = Yes), physical activity (0 = none; 1 = low; 2 = moderate; 3 = high), PSS, Stress_DayBefore_ (1 = Not at all, to 5 = Extremely), Stress_SameDay_ (1 = Not at all, to 5 = Extremely), sleep quality of the night preceding the saliva sample collection (1 = Quite poor, to 5 = Quite good), time of waking and mean sleep time the day of saliva sample collection. *Step 2 (predictors)* = In step 2 we included CAR or cortisol awakening as independent variables.

Moreover, Kumari et al. [7] showed that the relationship between the CAR and WS was especially observed in men. Therefore, we investigated whether the relationships observed were moderated by sex. To do so, in step 1 we added sex (0 = women; 1 = men) to the covariates previously included, and in step 3 we included the interaction between the CAR or cortisol awakening and Sex to investigate possible sex-related differences. Results showed that cortisol awakening was not related to WS (β = -0.101; *p* = 0.335) and that a CAR of reduced magnitude was still related to slower WS (β = -0.216; *p* = 0.029). However, none of these relationships were moderated by sex (*p*>0.785).

Finally, to reduce the possible effect of an inaccurate CAR measurement on the results, we excluded from the analyses the saliva sampling days that showed a negative CAR. This approach maximizes the ratio of properly measured CARs to CARs, but at the same time, it means that for 29% of the sample, only one day of CAR assessment is included in the analyses, increasing the possible effect of state factors on our results [26]. To control for these possible state effects, we explored whether the relationship between CAR and WS was still observed when participants showing a positive CAR on only one day were excluded from the analyses. Regression analyses showed a significant association between a CAR of reduced magnitude and slower WS (β = -0.271; *p* = 0.042). This association was marginally significant when sex (β = -0.224; *p* = 0.076) and cortisol awakening (β = -0.252; *p* = 0.089) were added to the covariates. It is possible that the reduction in the significance when sex and cortisol awakening are included as covariates may be due to a 29% reduction in the sample. Additionally, it is important to note that the *p* value reported is two-tailed, and given that these analyses are performed to confirm the previous results observed with the complete (and larger) sample, a one-tailed *p* value could also be considered. In this case, these associations would be statistically significant (*p*<0.05). The relationships between cortisol awakening and WS and the interaction between Sex and CAR or cortisol awakening remained non-significant (*p*>0.231). These results suggest that the associations observed are not driven by the subgroup of participants with positive CAR on only one day.

## Discussion

We observed that a CAR of reduced magnitude was related to worse performance on a WS task. This relationship was not moderated by sex. Our findings confirm the results observed by Gardner et al. [8] and Gardner et al. [9], but they contrast with those observed in Kumari et al. [7] and Johar et al. [10], who observed the opposite relationship and no relationship, respectively. It is worth noting that, compared to this previous research, in the present study we used three salivary samples on two consecutive weekdays to measure cortisol levels, we controlled for possible non-adherence to the protocol, enhancing the reliability of CAR measurements, and we controlled for perceived stress in the previous month and self-reported physical activity. Additionally, we show for the first time that the relationship between CAR and WS is independent from cortisol levels on awakening, an indicator of pre-awakening cortisol secretion that can affect the magnitude of the CAR [2].

Previous studies have shown that less diurnal variability in cortisol levels is related to slower WS [7, 8, 9, 10]. It has been suggested that a dysregulation of the HPA-axis activity may be a potential mechanism for sarcopenia (i.e., loss of muscle mass and strength), affecting physical performance and WS [8]. Thus, a possible explanation is that a CAR of reduced magnitude, along with higher night cortisol levels, could lead to less dynamic HPA-axis activity and less diurnal variability in cortisol levels, contributing to sarcopenia.

However, two other possible explanations can be considered. It is possible that the CAR affects physical performance, independently from the rest of the HPA-axis, because the CAR is considered a discrete component of HPA-axis activity that may make a separate contribution to a person’s health condition [1]. Along these lines, recent studies in young and older people have shown that a CAR of reduced magnitude is related to worse performance on executive function-related tasks [4, 27], a cognitive ability that seems to be critical in walking performance [28]. In older people, Evans et al. [4] observed that participants showing a CAR of reduced magnitude performed worse on the Trail Making Test B, a test used to assess the cognitive flexibility component of executive function. Similarly, in a 50-day case study, Law et al. [27] recently showed that a CAR of decreased magnitude predict worse performance on the Attention Switching Task (an executive function-related task) the same day. To date, no causality has been established between the CAR and cognitive performance, but it has been suggested that inter-individual differences in CAR provoke differences in executive function [27]. In addition to the relationship between CAR and executive function, previous studies have shown that there is a strong relationship between a high-order cognitive control mechanism and WS in older people [29–32]. Although not exclusively, executive function and attention play an important role in WS. Previous studies have observed that baseline executive function predicts a decline in gait performance [33–36], and that executive function and attention may be used to identify older adults at the greatest risk of mobility decline [33, 37]. Therefore, it is possible that changes in CAR might affect executive function, leading to worse walking performance. Further studies are needed to test whether executive function mediates the relationship between CAR and WS.

Another possible explanation is that the observed relationship between the CAR and WS would mean that both measures can function as markers for similar age-related changes and health conditions. Indeed, both a CAR of reduced magnitude and slow WS have been related to worse prefrontal cortex-related cognitive functioning and cardiovascular disorders [1, 4, 6].

Importantly, the results from our study and previous studies indicate that the association between CAR and WS is observed even when different tasks are used to measure WS, and they suggest that the CAR would be specifically related to the ability to walk. In this direction, Gardner et al. [9] reported that a CAR of decreased magnitude was related to slower WS, measured with the timed up-and-go test (TUG) (time needed to get up from a chair, walk 3m at normal speed, turn around, walk back and sit down is assessed), but no significant association was observed with the sit-to-stand test (time participants take to stand up from a chair and sit down as fast as possible). Together, these results suggest that the CAR would be specifically related to the ability to walk, but not to the time needed to stand up and sit down on a chair. This specific relationship may be due to the fact that, as observed for the CAR, the ability to walk shows a higher relationship with high-order cognitive control mechanisms (especially executive function) than the sit-to-stand test, a task that shows a stronger relationship with lower limb strength [38–40].

One limitation of this study is that, due to the cross-sectional design, no conclusions can be drawn about causality. In comparison with previous studies, the sample in the current study is relatively small. Although this might reduce the statistical power of our study, it allows us to include a larger number of salivary samples to measure the CAR (3 samples each day, for two consecutive days) and have greater control over our participants. Another limitation of the study is that we used only one trial to measure WS, an issue that might affect the reliability of the WS measurement. However, it should be noted that previous studies have shown a very high correlation between the performance on the first and second trials of the WS (*r*>0.90), and it has been suggested that one trial of the test may adequately represent subjects’ performance [41–43]. Finally, we objectively verified the timings of the second and third samples in relation to the first, but we did not include any objective measures (e.g., actigraphy, electroencephalography, polysomnography) to control for a possible delay between the time of waking and the provision of the first sample. We provided information and instructions to the participants to maximize the adherence to the saliva sampling protocol. Additionally, we controlled for a possible delay in the first salivary sample by exploring their cortisol patterns and excluding sampling days with a negative CAR. Although this approach does not guarantee that non-adherent CAR days are not included in the analyses, it reduces this possible effect. It is worth noting that we identify 14.5% days as the number of days with a possible delay in the first salivary sample, a percentage similar to the one shown in Griefahn and Roberns [44], who observed that there was a delay of more than 30min in the first salivary sample of 14% of 510 objectively measured CAR days. However, it is important to investigate whether these results are still observed when electronic devices to objectively verify the awakening time are used.

In conclusion, our study indicates that a CAR of reduced magnitude in older people is related to slower WS. This might imply that the CAR contributes to physical performance, independently from the rest of the HPA-axis. Some possible explanations might be that a CAR of reduced magnitude affects WS through a possible negative effect on executive function and/or that the CAR and WS could be considered as markers for similar age-related changes in health condition and/or cognitive performance. The relationship between CAR and physical performance should be studied more in depth because it might be considered as a separate intervention target to improve physical functioning in older people.
